# Distinct gene expression patterns for CD14++ and CD16++ monocytes in preeclampsia

**DOI:** 10.1038/s41598-022-19847-5

**Published:** 2022-09-14

**Authors:** Polina Vishnyakova, Maria Kuznetsova, Anastasiya Poltavets, Mariia Fomina, Viktoriia Kiseleva, Kamilla Muminova, Alena Potapova, Zulfiya Khodzhaeva, Alexey Pyregov, Dmitry Trofimov, Andrey Elchaninov, Gennady Sukhikh, Timur Fatkhudinov

**Affiliations:** 1 National Medical Research Center for Obstetrics, Gynecology and Perinatology Named After Academician V.I. Kulakov of Ministry of Healthcare of Russian Federation, Moscow, Russia; 2 Peoples’ Friendship University of Russia, Moscow, Russia; 3 A.P. Avtsyn Research Institute of Human Morphology, Moscow, Russia

**Keywords:** Mechanisms of disease, Molecular medicine, Inflammation

## Abstract

Preeclampsia (PE) is a serious gestational complication affecting the life of a mother and child. The immunophenotype and gene expression profile of isolated blood monocyte subpopulations of pregnant women with PE have not been studied before. In this work, we assessed changes in CD14++ and CD16++ monocyte subpopulations in PE and physiological pregnancy (n = 33). Immunophenotyping, immunomagnetic sorting of monocytes and analysis of the transcriptional profile of their genes were carried out. The percentage of classical monocytes was significantly lower, while the intermediate fraction of monocytes was significantly higher in late-onset PE compared to control. Transcriptome analysis of late-onset PE classical CD14++ monocytes revealed significant activation of inflammation mediated by chemokine and cytokine signalling pathways; apoptosis; regulation of transcription from RNA polymerase II promoter in response to stress and others. The most suppressed signalling pathways were associated with T cell activation and selection. In CD16++ monocytes of late-onset PE cases, positive regulation of cell–cell adhesion, integrin signalling pathway, blood coagulation cascade were the most activated ones. The inflammation mediated by chemokine and cytokine signalling pathway and p53 pathway were the most down-regulated in CD16++ monocytes. The obtained results indicate profound changes occurring to two most polar monocyte subpopulations in PE and their different roles in the pathogenesis of this disease.

## Introduction

Preeclampsia (PE) is a serious obstetric pathology remaining the leading cause of maternal and fetal morbidity and mortality^1^. PE complicates on average 8% of pregnancies and is characterized by hypertension in combination with proteinuria/fetoplacental or maternal organ dysfunction manifesting after 20 weeks of gestation. Monocyte–macrophage lineage plays an important role in maintaining physiological pregnancy since macrophages, along with NK cells, are a major cell population in the decidua basalis, the maternal part of the placenta^2^. Monocytes originate in the bone marrow and give rise to tissue macrophages. In humans, they are divided into two main subpopulations: classical (CD14++) and nonclassical (CD16++) monocytes, depending on the expression of the mentioned above markers on their surface^3^. Researchers also distinguish an intermediate subpopulation that carries both markers (CD14++CD16+). CD14 is a membrane protein component of the lipopolysaccharide receptor complex. CD16 is the FcγRIII receptor responsible for antibody-dependent cellular cytotoxicity^4,5^. The functional differences between these two subpopulations are now being actively studied. Classical monocytes are the main sources of the macrophage pool in tissues. It is believed that non-classical monocytes are responsible for vascular patrolling by scanning the surface of the endothelium in search of pro-inflammatory signaling molecules^6^. Functional differences in monocytes are emphasized by their different responses to pro-inflammatory stimuli^7,8^. Plasma levels of pro-inflammatory cytokines are increased in several pathologies, in particular in PE: elevated concentrations of different cytokines represent a laboratory feature accompanying PE that has also been demonstrated in our previous study and other works^9–11^. These changes in the plasma cytokine profile certainly could affect monocytes functions.

It can be assumed that a change in the profile of monocytes, their phenotype, and the genes they express could affect those organs where monocytes come from the bloodstream, in particular the placenta. The aim of our work was to evaluate what happens to the two most polar subpopulations of peripheral blood monocytes in PE compared to physiological pregnancy.

## Results

### Patients’ data

By gestational age at diagnosis, PE is commonly classified into early-onset PE (eoPE) and late-onset PE (loPE) manifesting before or after 34 weeks of gestation, respectively. In present study all patients were divided into three groups based on clinical data (Table 1): control (n = 11), eoPE (n = 12) and loPE (n = 10). As expected, gestational age at delivery and birthweight were significantly lower in PE groups compared to control (p < 0.01), whereas systolic and diastolic blood pressures were significantly increased in PE groups compared to control (p < 0.01). Proteinuria was detected in PE groups only.

**Table 1:** Demographic and clinical characteristics of the patients.

Characteristics	Control	eoPE	loPE
Group size (n)	11	12	10
Maternal age, years	32.2 ± 1.1	32.3 ± 1.7	32.6 ± 1.6
Gestational age at delivery, weeks	38.5 ± 0.2	30.5 ± 1.0*	36.8 ± 0.3
Systolic blood pressure, mmHg	115.6 ± 2.1	160.5 ± 5.2*	144.8 ± 4.6*
Diastolic blood pressure, mmHg	73.1 ± 1.5	104.1 ± 5.1*	93.3 ± 3.6*
Proteinuria, g/L	nd	2.4 ± 0.6	2.0 ± 0.7
Anesthesia during labor	Epidural	Epidural	Epidural
Newborn mass, g	3484.5 ± 145.4	1337.5 ± 165.2*	2474.7 ± 113.5*
Sex of the newborn (Male/Female)	5/6	5/7	3/7
Intrauterine growth restriction, number of cases	0	3	3

The data are listed as mean ± SEM; *p < 0.01 vs control; *nd* not detected.

### Monocyte profiles

**Figure 1:**
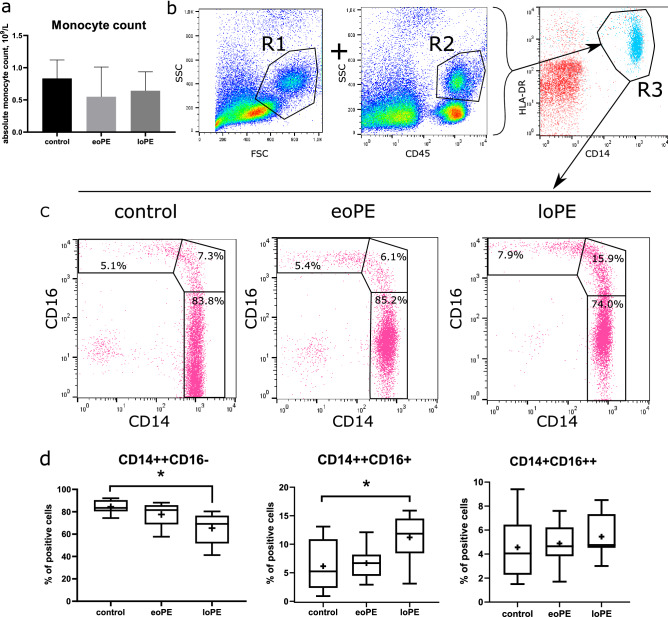
Characterization of the patient's monocytes. Absolute monocyte counts for three groups of patients according to clinical diagnostic laboratory reports: the data are listed as mean ± SD (**a**). In flow cytometry analysis, monocytes were defined by sequential gating: cells that were found in both regions of interest R1 on FSC-SSC dot-plot and R2 on SSC-CD45 dot-plot, then were displayed and gated on CD14 versus HLA-DR dot-plot (R3) to exclude B lymphocytes (**b**). The selected population was analyzed in CD14–CD16 dot-plots. Representative CD14–CD16 dot-plots showing classical (CD14++CD16−), intermediate (CD14++CD16+), and non-classical (CD14+CD16++) monocytes in upper-left, middle and bottom-right regions, respectively (**c**). Boxplots of classical, intermediate, and non-classical monocyte content for three groups (**d**): *eoPE* early-onset PE (n = 12), *loPE* late-onset PE (n = 10), control (n = 11). *p < 0.05 according to Kruskal–Wallis test with Dunn's post-hoc test; box limits are IQRs, horizontal bars are medians, and crosses are means.

**Figure 2:**
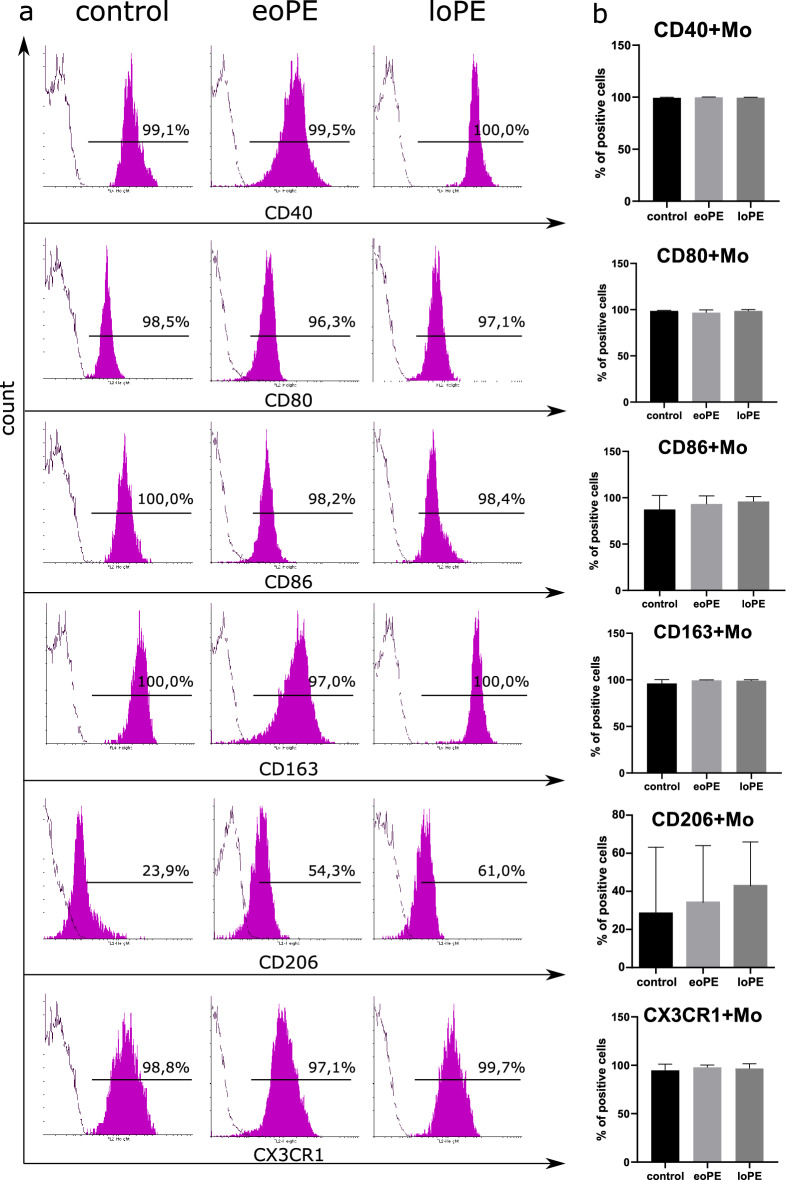
Immunophenotyping of the patient's monocytes. Representative histograms of gated monocytes stained with antibodies to CD40, CD80, CD86, CD163, CD206 or CX3CR1 (magenta-filled histogram) and control samples (empty contour) for three groups of patients (**a**). Percents of positively stained cells are indicated above the gate bars. Levels of CD40+, CD80+, CD86+, CD163+, CD206+ and CX3CR1+ monocytes in three groups: the data are listed as mean ± SD (**b**): *eoPE* early-onset PE (n = 12), *loPE* late-onset PE (n = 10), control (n = 11).

The first step of our study was to compare the absolute number of monocytes according to clinical diagnostic reports. There were no significant differences in monocyte count between the groups (Fig. 1a). Then we performed general monocyte phenotyping before their isolation by magnetic sorting. Monocytes were gated both in dot-plot of forward and side scatter (FSC-SSC, R1 in Fig. 1b) and SSC-CD45 dot-plot (R2 in Fig. 1b) with subsequent gating on CD14-HLA-DR dot-plot (R3 in Fig. 1b). After that, we determined the percentage of cells stained positively for CD14 and CD16 markers. Significant differences were demonstrated for classical and intermediate monocyte fractions (Fig. 1c, left and middle plots in Fig. 1d:), with the percent of CD14++CD16− monocytes decreased (p = 0.003) and the percent of CD14++CD16+ monocytes increased (p = 0.03) in loPE group compared to control. We found no differences in non-classical (CD14+CD16++) monocytes level between the studied groups. The content of monocytes stained positively for CD80, CD40, CD86, CD163, CX3CR1, and HLA-DR markers was similar between the groups and close to 100% (Fig. 2). The content of CD206+ monocytes in control group was lower compared to PE groups, albeit non-significantly. As significant differences in monocyte composition were demonstrated only in loPE group, we used monocytes sorted by CD14 and CD16 markers only from loPE and control patients for subsequent microarray analysis. Monocytes were sorted using anti-CD14 and anti-CD16 magnetic beads; each fraction was stained with anti-CD14 and anti-CD16 antibodies, respectively, to determine the purity of subpopulations, which reached ≥ 90% (Fig. S1 in Supplementary Material).

### Gene expression profiles of loPE CD14++ monocytes vs control CD14++ monocytes

**Figure 3:**
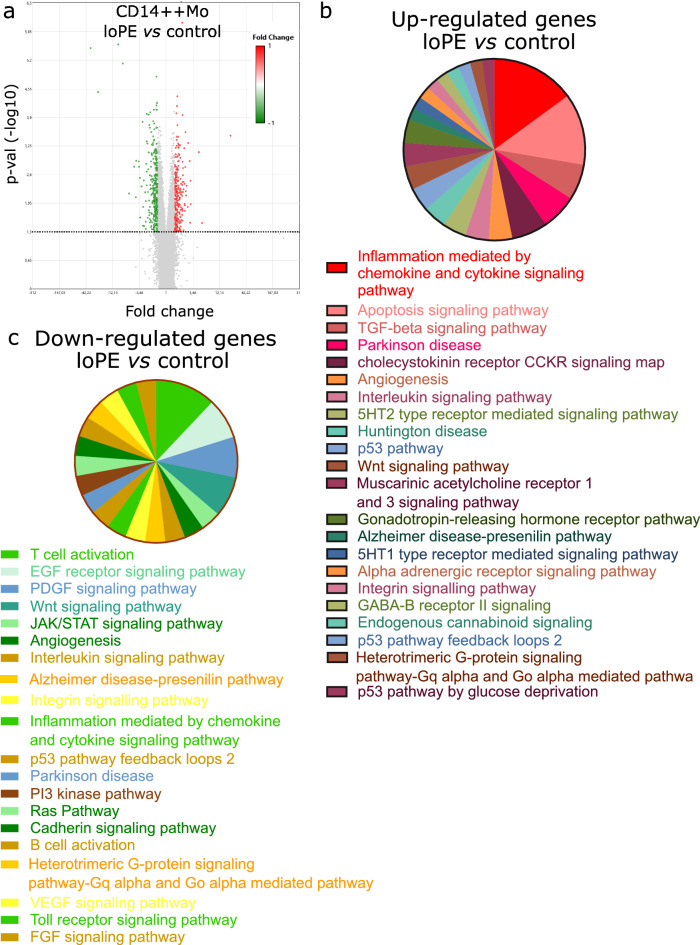
Transcriptomic data (relative gene expression levels) for CD14++ blood monocytes of late-onset PE (loPE) and control groups. Volcano plot (**a**) provides a comparative overview of gene expression in CD14++ monocytes of loPE (n = 6) vs control group (n = 7). Each point in the plot corresponds to a gene. The red- and green-colored genes positioned above the horizontal dotted line are significantly (p < 0.05) up- or down-regulated in loPE CD14++ cells, respectively. The data were analyzed with TAC Software 4.0.2 using Affymetrix default analysis settings and RMA algorithm. Enrichment analysis for upregulated genes (**b**) and downregulated genes (**c**) grouped in the PANTHER ontology by molecular pathway and listed in order starting from the most represented.

Next we performed immunomagnetic separation of monocytes from peripheral blood mononuclear cells (PBMC) collected from patients of loPE and control groups. According to our previous experience of sorting with anti-CD14/CD16 beads, the yielded CD14++ isolates are mostly classical monocytes, whereas CD16++ isolates (further denoted as CD16++ monocytes) are mostly non-classical monocyte subpopulations with a fraction of intermediate monocytes^7^ (Fig. S2). Comparative microarray hybridization for loPE vs control revealed 580 differentially expressed transcripts in sorted CD14++ monocytes (p < 0.05), including protein-coding and long intergenic non-coding RNAs. Figure 3a shows transcripts found to be at least 1.5-fold up- or down-regulated (red and green points, respectively) in loPE CD14++ monocytes compared to control group. The most pronouncedly hyperexpressed in loPE group were several long intervening/intergenic noncoding RNAs (lincRNAs): linc-FCGR1B-7, linc-RAB23-2, linc-PARD6G-2, and linc-POTED-9. The most down-regulated sequences in loPE group included TRAV20 (T cell receptor alpha variable 20), TRAJ35 (T Cell Receptor Alpha Joining 35), and ADGRE4P (Adhesion G protein-coupled receptor E4P). The list of ten most up- and down-regulated transcripts is given in Table S1 (see Supplementary Material). We used PANTHER software to classify the up- and down-expressed genes by specific pathways (Fig. 3b), biological processes, and molecular functions (Table 2; Table S2, Supplementary Material). Molecular pathways activated in loPE CD14++ monocytes included: inflammation mediated by chemokine and cytokine signaling pathway (*JUN*,* GNA11*,* CXCR1*,* CXCL8*,* CCL3L3*,* CCL3*,* CCL4L1*); apoptosis signaling pathway (*TP53*,* HSPA6*,* HSPA1A*,* TNFRSF10C*,* JUN*,* HSD17B1*); TGF-beta signaling pathway (*CITED2*,* CITED4*,* JUN*); Parkinson disease (*HSPA1B*,* HSPA1A*,* HSPA6*); and cholecystokinin receptor (CCKR) signaling map (*ODC1*,* CXCL8*,* JUN*) (Fig. 3b). The PANTHER classification of hyperexpressed transcripts by biological processes and molecular functions revealed over tenfold enrichment in loPE CD14++ monocytes for regulation of transcription from RNA polymerase II promoter in response to stress: regulation of DNA-templated transcription in response to stress (*PSMB6*,* HSPA1A*,* SESN2*,* CITED2*,* JUN*,* TP53*); protein deubiquitination: thiol-dependent deubiquitinase; cysteine-type endopeptidase activity; omega peptidase activity; and ubiquitin-like protein-specific protease activity (*USP17L22*,* USP17L5*,* SPATA2*,* USP17L6P*,* PSMB6*,* USP17L18*,* USP17L12*,* USP17L20*,* USP17L21*,* USP17L25*,* USP47*,* USP17L17*,* USP17L19*,* USP17L11*,* AMZ2*,* MME*,* DPP3*,* NLRP1*) (Table 2). Among the down-regulated genes, the most suppressed in loPE CD14++ monocytes were involved in the following pathways: T cell activation, T cell selection, positive regulation of interferon-gamma production, negative regulation of natural killer cell-mediated immunity (*IL7R*,* CD2*,* CD28*,* CD3E*,* TXK*,* IKZF3*,* SLAMF6*,* THEMIS*,* RASGRP1*,* MAL*,* GPR18*,* ITK*,* LEF1*,* KLRC4*,* CD96*); EGF and PDGF receptor signaling pathways (*KRAS*,* STAT4*); Wnt signaling pathway (*PPP3CC*,* LEF1*) (Fig. 3c; Table S2, Supplementary Material).

**Table 2:** Enrichment analysis for genes significantly upregulated in CD14++ monocytes of loPE vs control groups performed in PANTHER.

	Signaling pathway	Fold enrichment	p-value	FDR
Biological process	Regulation of transcription from RNA polymerase II promoter in response to stress (GO:0043618)	11.63	2.05E−05	1.79E−02
	Regulation of DNA-templated transcription in response to stress (GO:0043620)	10.63	3.29E−05	2.45E−02
	Protein deubiquitination (GO:0016579)	10.59	1.76E−10	2.76E−06
	Protein modification by small protein removal (GO:0070646)	9.54	6.29E−10	4.93E−06
	Modification-dependent protein catabolic process (GO:0019941)	3.58	1.46E−05	1.76E−02
	Cellular macromolecule catabolic process (GO:0044265)	3.57	8.98E−08	2.01E−04
	Modification-dependent macromolecule catabolic process (GO:0043632)	3.51	1.84E−05	1.92E−02
	Macromolecule catabolic process (GO:0009057)	3.47	4.38E−08	1.72E−04
	Proteolysis involved in cellular protein catabolic process (GO:0051603)	3.44	1.29E−05	1.69E−02
	Ubiquitin-dependent protein catabolic process (GO:0006511)	3.42	4.47E−05	3.19E−02
	Cellular protein catabolic process (GO:0044257)	3.28	2.29E−05	1.89E−02
	Protein catabolic process (GO:0030163)	3.19	1.87E−05	1.83E−02
	Regulation of programmed cell death (GO:0043067)	2.82	6.78E−08	1.77E−04
	Regulation of apoptotic process (GO:0042981)	2.79	1.41E−07	2.76E−04
	Cellular catabolic process (GO:0044248)	2.75	2.93E−08	1.53E−04
	Regulation of cell death (GO:0010941)	2.64	1.93E−07	3.36E−04
	Catabolic process (GO:0009056)	2.55	4.90E−08	1.54E−04
	Organic substance catabolic process (GO:1901575)	2.54	7.31E−07	1.15E−03
	Macromolecule metabolic process (GO:0043170)	1.55	1.94E−05	1.78E−02
	Cellular metabolic process (GO:0044237)	1.47	2.49E−05	1.95E−02
	Metabolic process (GO:0008152)	1.46	4.13E−06	5.88E−03
	Primary metabolic process (GO:0044238)	1.46	4.78E−05	3.26E−02
	Organic substance metabolic process (GO:0071704)	1.45	1.57E−05	1.76E−02
Molecular function	Thiol-dependent deubiquitinase (GO:0004843)	12.72	5.34E−10	2.61E−06
	Deubiquitinase activity (GO:0101005)	12.51	6.38E−10	1.56E−06
	Cysteine-type endopeptidase activity (GO:0004197)	12.2	8.30E−10	1.36E−06
	Omega peptidase activity (GO:0008242)	11.28	1.91E−09	2.33E−06
	Ubiquitin-like protein-specific protease activity (GO:0019783)	11.19	2.06E−09	2.02E−06
	Cysteine-type peptidase activity (GO:0008234)	7.52	1.30E−07	1.06E−04
	Carbohydrate binding (GO:0030246)	4.53	9.99E−05	4.08E−02
	Endopeptidase activity (GO:0004175)	4.12	5.47E−06	3.83E−03
	Peptidase activity (GO:0008233)	3.46	6.52E−06	3.99E−03
	RNA binding (GO:0003723)	2.13	1.05E−04	3.97E−02
	Hydrolase activity (GO:0016787)	1.96	5.05E−05	2.25E−02

### Gene expression profiles of CD16++ monocytes (loPE vs control)

**Figure 4:**
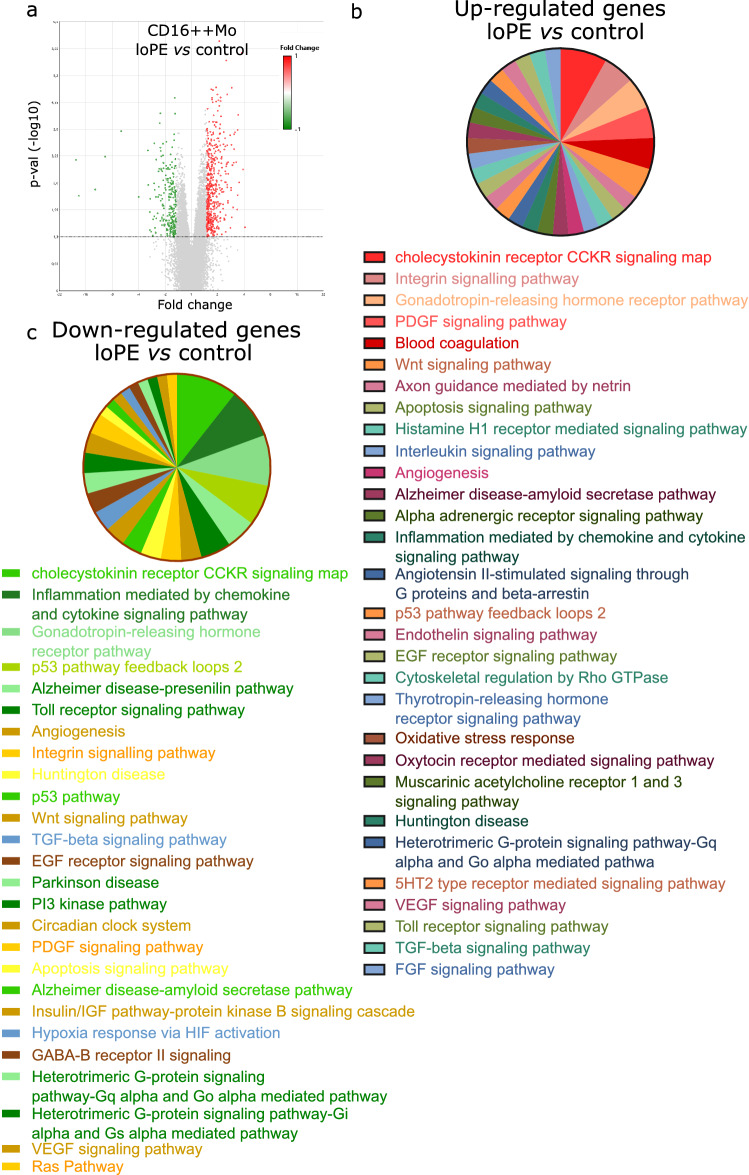
Transcriptomic data (relative gene expression levels) for CD16++ blood monocytes of late-onset PE (loPE) and control groups. Volcano plot (**a**) provides a comparative overview of gene expression of CD16++ monocytes of loPE (n = 6) vs control group (n = 6). Each point in the plot corresponds to a gene. The red- and green-colored genes positioned above the horizontal dotted line are significantly (p < 0.05) up- or down-regulated in loPE CD14++ cells, respectively. The data were analyzed with TAC Software 4.0.2 using Affymetrix default analysis settings and RMA algorithm. Enrichment analysis for upregulated genes (**b**) and downregulated genes (**c**) grouped in the PANTHER ontology by molecular pathway and listed in order starting from the most represented.

Microarray gene expression analysis of CD16++ monocytes from patients with loPE vs control group revealed significant representation differences for 758 transcripts (Fig. 4a). Similarly with the corresponding analysis for CD14++ cells, the most heavily up-regulated were lincRNAs including linc-RAB23-2, linc-PARD6G-2, linc-FCGR1B-7, and linc-POTED-9. The down-regulated genes included the oxidized low-density lipoprotein receptor 1 (OLR1), linc-BOLA2, and small nucleolar RNA C/D box 56B (SNORD56B). The list of ten most up- and down-regulated transcripts is given in Table S3 (see Supplementary Material). Enrichment analysis identified cholecystokinin receptor (CCKR) signaling map, PDGF signaling pathway (*MYC*,* PRKCA*,* CLU*), integrin signalling pathway, blood coagulation (*ITGA2B*,* ITGB3*), gonadotropin-releasing hormone receptor pathway (*PRKCA*,* SNRPN*) as the most up-regulated (Fig. 4b). Classification by biological processes identified erythrocyte development (*ALAS2*,* DMTN*,* CITED2*,* SLC4A1*), protein de-ubiquitination and protein modification by small protein removal (*USP17L22*,* USP17L5*,* USP17L6P*,* USP17L18*,* USP17L12*,* USP17L20*,* USP17L21*,* USP17L25*,* USP17L15*,* USP17L17*,* USP17L19*,* USP17L11*), and positive regulation of cell–cell adhesion (*TESPA1*,* CD5*,* IL7R*,* CD27*,* TMIGD2*,* CCR7*) as the most over-represented (Table 3). Enrichment analysis for down-regulated genes was successfully performed for molecular pathways only (Fig. 4c). And again, cholecystokinin receptor (CCKR) signaling map (*PTEN*,* PTGS2*,* CSNK1D*,* CTNNB1*,* CXCL2*,* NFKBIA*) and gonadotropin-releasing hormone receptor pathway (*SKIL*,* MAP3K8*,* CTNNB1*,* RAP1B*,* PER1*) were observed among the most suppressed pathways alongside the inflammation mediated by chemokine and cytokine signaling pathway (*PTEN*,* PTGS2*,* C5AR1*,* KRAS*,* NFKBIA*) and p53 pathway feedback loops 2 (*PTEN*,* CTNNB1*,* E2F3*,* KRAS*)*.*

**Table 3:** Enrichment analysis for genes significantly up-regulated in CD16++ monocytes of loPE vs control groups performed in PANTHER.

	Signaling pathway	Fold enrichment	p-value	FDR
Biological process	Erythrocyte development (GO:0048821)	20.07	6.83E−05	4.87E−02
	Protein deubiquitination (GO:0016579)	11.02	4.34E−08	6.80E−04
	Protein modification by small protein removal (GO:0070646)	9.93	1.10E−07	8.59E−04
	Positive regulation of cell–cell adhesion (GO:0022409)	5.55	4.50E−05	4.15E−02
	Positive regulation of leukocyte activation (GO:0002696)	4.54	3.82E−05	4.27E−02
	Adaptive immune response (GO:0002250)	4.49	6.71E−07	2.10E−03
	Positive regulation of cell activation (GO:0050867)	4.39	5.13E−05	4.24E−02
	Ubiquitin-dependent protein catabolic process (GO:0006511)	4.32	1.22E−05	2.12E−02
	Modification-dependent protein catabolic process (GO:0019941)	4.23	1.49E−05	2.34E−02
	Modification-dependent macromolecule catabolic process (GO:0043632)	4.15	1.83E−05	2.39E−02
	Regulation of cell activation (GO:0050865)	3.9	8.07E−06	1.58E−02
	Proteolysis involved in cellular protein catabolic process (GO:0051603)	3.83	4.17E−05	4.09E−02
	Regulation of leukocyte activation (GO:0002694)	3.66	6.51E−05	5.10E−02
	Cellular protein catabolic process (GO:0044257)	3.65	6.61E−05	4.94E−02
	Protein catabolic process (GO:0030163)	3.61	3.82E−05	3.99E−02
	Positive regulation of immune system process (GO:0002684)	3.29	1.74E−05	2.48E−02
	Immune response (GO:0006955)	2.99	4.10E−07	1.61E−03
	Regulation of immune system process (GO:0002682)	2.87	4.51E−06	1.01E−02
	Regulation of cell death (GO:0010941)	2.83	2.00E−06	5.23E−03
	Regulation of apoptotic process (GO:0042981)	2.67	3.60E−05	4.34E−02
	Regulation of programmed cell death (GO:0043067)	2.61	4.81E−05	4.19E−02
	Immune system process (GO:0002376)	2.55	3.03E−07	1.58E−03

### Effects of blood plasma (control vs loPE) on CD14++ monocytes isolated from matched healthy donors

**Figure 5:**
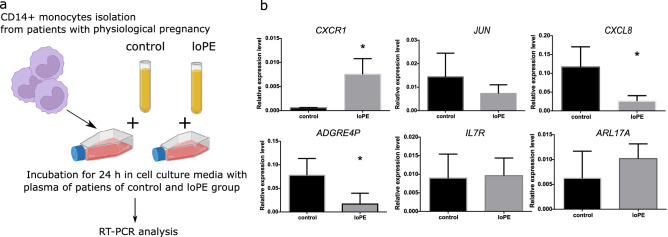
Effects of blood plasma (control vs loPE) on CD14++ monocytes isolated from healthy pregnant gestational age-matched donors (n = 3). A scheme of the experiment (**a**). CD14++ monocytes isolated from pregnant gestational age-matched donors were incubated in culture medium supplemented with plasma (10%) obtained from control (n = 3) or loPE (n = 3) patients for 24 h followed by RNA isolation and RT-PCR analysis (**b**). *loPE* late-onset PE. *p < 0.05 according to Mann–Whitney test. The data are presented as mean ± SD.

We further set to figure out whether the observed changes in monocyte expression profiles in loPE patients were induced by an exogenous source (e.g. by the plasma cytokines) or occurred autonomously through realization of intrinsic program. We exposed the sorted CD14++ monocytes from gestational age-matched pregnant donors to cell culture media supplemented with blood plasma obtained from loPE vs control groups (Fig. 5a). The cultures were harvested for RT-PCR analysis of genes found to be the most up- (*CXCR1*,* JUN*,* CXCL8*) or down- (*ADGRE4P*,* IL17R*, *ARL17A*) regulated according to microarray experiments (Table S1, Supplementary Material). We observed a significant increase in *CXCR1* expression by CD14++ monocytes in response to incubation with loPE plasma samples (Fig. 5b), whereas *JUN* expression levels were similar between the groups. The loPE plasma exposure promoted a decrease in *CXCL8* expression by CD14++ monocytes, contrary to expectations based on microarray data. Significant decrease for *ADGRE4P*, was consistent with CD14++ monocyte profiles for loPE patients (Table S1, Supplementary Material). For *IL17R* and *ARL17A*, expression levels were similar between the groups.

## Discussion

Recent decades were marked by intensive and comprehensive research on PE and its immunological aspects, but no decisive contributions to the field of therapy and prediction of this severe pregnancy complication. PE continues to be the leading cause of maternal and perinatal mortality and morbidity worldwide. The involvement of the monocyte–macrophage system in PE was confirmed by many experimental studies demonstrating an increase in pro-inflammatory and a decrease in anti-inflammatory macrophage numbers in the placenta, both fetal and maternal parts of the organ^10,12–16^. A possible role of blood monocytes as macrophage precursors in PE remains an open issue. A shifted balance of maternal blood monocyte subpopulations in PE, towards the predominance of non-classical or intermediate subpopulations, has been demonstrated. Our results are consistent with these findings. However, the immunophenotype is only one of the facets of monocytes. In this work, we for the first time report PE-associated changes of subset-specific gene expression signatures in CD14++ and CD16++ monocytes.

The imbalanced ratio of classical, intermediate, and non-classical macrophage subpopulations is a hallmark of PE. Such imbalances are known to be more indicative of particular physiological or pathological processes than the total monocyte population dynamics^17,18^, which was also confirmed for PE. In this study, we observe a significant decline in relative counts of classical monocytes and a concomitant increase in counts of intermediate monocytes in patients with loPE. This observation agrees with other published evidence on the increased proportion of intermediate and non-classical monocytes in PE^19,20^. As reported by Alahakoon et al., PE with or without intrauterine growth restriction (IUGR) is accompanied by significantly lower percentage of classical monocytes, but boosted inflammatory monocyte subsets (intermediate and non-classical) compared to normal pregnancy^21^. These data are consistent with a study by Jabalie et al. observing a decline of classical monocytes alongside increased counts of intermediate and non-classical monocytes in PE^22^. The ontogenetic relationship between monocyte subpopulations and their role in homeostasis remains a subject of intensive research^17,23^. It is currently assumed that monocytes of the intermediate subpopulation represent an intermediate stage of monocyte differentiation: the classical bone marrow monocytes migrate to vessels and differentiate first into CD14++CD16+ (intermediate) and then into CD14+CD16++ (non-classical)^24^. The non-classical CD14+CD16++ monocytes function as patrol cells that regulate endothelial repair^25^. Considering the association of PE with endothelial dysfunction, the increase in CD16++ monocyte numbers can be viewed as reflecting the process of endothelial repair stimulation under conditions of endothelial damage in PE.

Immunophenotyping by flow cytometry revealed no PE-related differences in CD80, CD40, CD86, CD163, CD206, CX3CR1 and HLA-DR expression by monocytes. However, Nunes et al. describe down-regulation of scavenger receptor CD163 on monocytes in patients with PE. This inconsistency can be explained by the use of different assay: the authors analyzed not the percentage of CD163+ monocytes but the CD163’s mean of fluorescence intensity of CD14-positive cells after short-time cell adhesion to culture plastic^26^.

For subsequent transcription profiling, two gestational age-matched groups were selected—loPE and control. We performed immunomagnetic sorting of PBMC by CD14 and CD16 markers, followed by gene expression microarray hybridization and enrichment analysis. The routine procedure of CD14++ monocyte isolation yields the classical subset constituting about 80–90% of total blood monocytes^7,27^. Classical monocytes are the main source of macrophages to which they give rise after migrating into the tissue for bridging innate and adaptive immune responses. Functionalities of classical monocytes include phagocytosis, antibody-dependent cell-mediated cytotoxicity, participation in coagulation, and cytokine secretion^28–30^. Our data indicate that, in loPE, CD14++ monocytes are subject to stress and catabolic restructuring, given that the up-regulated signaling pathways were mostly associated with chemokine- and cytokine-mediated inflammation, stress response, macromolecular changes in cell catabolism, ubiquitinylation/de-ubiquitinylation, apoptosis, and intracellular proteolysis. These inferences are consistent with other studies. Matias et al. demonstrate higher endogenous activation of NLRP1/NLRP3 inflammasomes responsible for caspase activation and TLR4/NF-κB pathway up-regulation in PE, as well as higher gene/protein expression for IL-1β, IL-18, and TNF-α^31,32^. In a recent study Xu et al. show that CD14+ monocytes from patients with PE produce higher levels of TNF-α, IL-1β, and IL-6 compared to normotensive pregnant women and associate these observations with down-regulation of α7 nicotinic acetylcholine receptor^33^. Interestingly, according to our results, most of the significantly suppressed signaling pathways were associated with T lymphocyte activation or differentiation. It is difficult to explain the reason for such changes in the transcriptome of loPE CD14++ monocytes. The decrease in the activity of signaling pathways associated with T lymphocytes probably occurs as a compensatory response to avoid their hyperactivation in PE. Still, there is no data unequivocally confirming this hypothesis. At early stages of lung cancer, as well as in B cell non-Hodgkin lymphoma, CD14+ monocytes exert immunosuppressive properties, which allow them to suppress the cytotoxic activity of T-lymphocytes^34,35^. A similar effect has been observed in Kawasaki disease characterized by pronounced immune mobilization, activation of monocytes and neutrophils, and elevated synthesis of IL-1, IL-6, and TNFα^36^. The elevated plasma levels of pro-inflammatory cytokines in Kawasaki disease are accompanied by elevated levels of anti-inflammatory cytokine IL35, which suppresses the ability of CD14+ monocytes to activate naive CD4+ T cells and has a protective effect^36^.

Consistently with our previous experience, CD16++ monocyte isolation procedure yields a mixture of the intermediate and non-classical monocyte subsets^7^, both of them involved in antigen processing and promotion of neutrophil adhesion to endothelium through TNFα production, and also specifically associated with wound healing processes^37,38^. In loPE, CD14++ and CD16++ monocytes differ by gene expression profiles, having only few signaling pathways in common. Signaling pathways up-regulated in CD16++ monocytes are associated with intercellular adhesion, cell-to-matrix adhesion, leukocyte activation, blood coagulation, apoptosis, and ubiquitinylation/de-ubiquitinylation. Several pathways are found in both the ‘activated’ and ‘suppressed’ enrichment lists, notably cholecystokinin receptor (CCKR) signaling map and gonadotropin-releasing hormone receptor pathway, which may indicate their participation in a complex bilateral crosstalk, as both are involved in cell survival, angiogenesis, and invasion^39^. Interestingly, the inflammatory cascade mediated by chemokine and cytokine signaling pathway has been identified as one of the most inhibited. These results suggest that, in loPE, the activated CD16++ monocytes follow their native program of vascular patrolling and innate immunity activation, which is consistent with their increased maternal blood counts^21,22,40^. The results indicate pronounced differences in functional activity and molecular signatures of CD14++ and CD16++ monocytes, particularly in loPE. Interestingly, four lincRNAs (linc-FCGR1B-7, linc-RAB23-2, linc-PARD6G-2 and linc-POTED-9) are found among the most up-regulated transcripts in both CD14++ and CD16++ monocytes of patients with loPE as compared to control group. The involvement of lincRNAs in pathogenic processes has not been studied in detail. Linc-FCGR1BP affiliates with a Fc Receptor *FCGR1BP* pseudogene apparently encoding a non-functional protein non-detectable at the cell surface and binding the ligand with low affinity^41^. Linc-PARD6G-2 is downregulated in multiple myeloma resistant to proteasome inhibitors^42^. Specific increase in lncRNA expression in PE, observed in several studies, places lncRNAs as prospective markers of PE. However, the exact list of lncRNAs up- and down-regulated in PE is controversial^43,44^, probably due to heterogeneity of PE and other factors that require further investigation. The nature of these lincRNA activation in monocytes in loPE remains obscure.

Our results raise the question of whether such molecular signature of CD14++ and CD16++ monocytes persists upon entering peripheral tissues or whether the cells undergo apoptosis. Monocytes are known to lose a number of markers (e.g. Ly6C in mice) upon infiltration and differentiation into macrophages^45^, but whether specific activation of pro-inflammatory and ubiquitinylation-related pathways persists after monocyte migration is unclear^17^. Several studies based on animal models suggest that monocytes extravasating constitutively into lymph nodes and tissues retain their transcriptional profiles and may even remain monocytes^23,46^. In addition, the activated monocytes may provide a source of massive cytokine secretion and thus modulate inflammation in the body^30,47,48^. The subset-specific gene expression signatures of monocytes in loPE may reflect the exposure to signaling agents (cytokines, chemokines, free nucleic acids, placental exosomes) circulating in maternal blood at elevated levels confirmed by multiple studies^49–53^. This hypothesis is partially supported by our data: expression of several genes up- and down-regulated in monocytes was differentially modified by exposure to blood plasma of patients with PE vs control group.

Overall, our study strengthens the concept of maternal immunological stress as a major contribution to PE, tracked by altered immunophenotypes and transcriptomic signatures of blood monocytes.

## Methods

### Ethics declarations

All experiments with blood were conducted in accordance with the Declaration of Helsinki, guidelines for Good Clinical Practice and Commission of Biomedical Ethics at the Research Center for Obstetrics, Gynecology and Perinatology, Ministry of Healthcare of the Russian Federation. All experimental protocols were approved by the Commission of Biomedical Ethics at the National Medical Research Center for Obstetrics, Gynecology and Perinatology of the Ministry of Healthcare of Russian Federation, Moscow (Ethic's committee approval protocol No. 5, 27th of May 2021). All participants signed informed consent in accordance with the Ethics Committee requirements and Helsinki Declaration of the World Medical Association.

### Clinical laboratory tests

Monocyte count measurements were performed on the Sysmex XS-800i Hematology Analyzer, according to the manufacturer's recommendations. The unit of measurement of monocytes is 10^9^/L with a reference interval of 0.09–0.8 × 10^9^/L. Measurement of protein concentration in urine samples was performed first by dipstick test then in case of positive result by the spectrophotometric method on an automatic biochemical analyzer BA-400 (Biosystems) using reagents and calibration references from the same manufacturer.

### Sample collection

Peripheral venous blood samples were collected prior to the caesarian section in EDTA-treated tubes (BD Vacutainer^®^ Plus). PBMC and monocytes were isolated from a single donation of 15 mL of whole blood. Plasma samples were obtained by centrifugation at 2000*g* at 4 °C for 15 min. The plasma samples were aliquoted under sterile conditions and stored at − 80 °C until experiment.

Isolation of CD14++ and CD16++ monocytes was performed using magnetic sorting with beads to CD14 and CD16 receptors as was previously described^7^. Briefly, blood was mixed in a 1:1 ratio with autoMACS^®^ Rinsing Solution (Miltenyi Biotec, Germany), and subjected to gradient centrifugation on Lympholyte-H Cell Separation Media (Cedarline, Canada) at 800*g* at room temperature for 20 min. The mononuclear cell fraction was collected and washed twice with Rinsing Solution (300*g* at 11 °C for 20 min). At this stage, an aliquot (100,000 cells) of PBMC was collected for further flow cytometry analysis. The rest of the cells were subjected to immunomagnetic sorting on a manual MidiMACS™ Separator by using LS, LD columns and magnetic anti-CD14, anti-CD16 beads (Miltenyi Biotec, Germany) in accordance to the manufacturer's protocols. To obtain CD14++ monocytes, positive selection using CD14 magnetic beads was performed. CD16++ monocytes were obtained using negative selection with FC-blocking reagent and depletion cocktail (the stage of depletion of granulocytes and NK cells), and further positive selection of pre-enriched target cell fraction using CD16 magnetic beads. The purity of each CD14++ and CD16++ monocyte sample was at least 90% (Figs. S1, S2). Cell count and viability was assessed on a TC20 analyzer (Bio-Rad, USA). For the experiment with the patient's plasma CD14++ monocytes were placed in 6-well cell-culture plates (Costar, USA), at a density of 0.5 × 10^6^ cells per well and incubated for 24 h in cell medium (RPMI supplemented with 2 mM l-glutamine, 25 U/mL penicillin, 25 μg/mL streptomycin and 10% patient's plasma instead of FBS).

### Flow cytometry analysis

For surface immunophenotype marker staining, the cells we resuspended in autoMACS^®^ Rinsing Solution (1 × 10^5^ cells in 100 µL) with 1% BSA and stained with anti-CD16 (A07766, Beckman Coulter), anti-CD14 (130-110-518, Miltenyi Biotec), anti-CD45 (A07785, Beckman Coulter), anti-CD206 (130-095-131, Miltenyi Biotec), anti-CD86 (130-116-160, Miltenyi Biotec), anti-CD163 (130-097-630, Miltenyi Biotec), anti-HLA-DR (130-111-790, Miltenyi Biotec), CD40 (130-110-947, Miltenyi Biotec), CD80 (130-117-683, Miltenyi Biotec) and anti-CX3CR1 (12-6099-42, eBioscience). The analysis was carried out on the FACScan flow cytometer (Becton Dickinson, USA) with the CellQuest software. In each measurement, 10,000 cells were analyzed. The gating strategy included selection of the monocyte population on dot-plot of forward (FSC) and side scatter (SSC), as well as gating on SSC vs CD45 flow dot-plot with further gating on CD14 vs HLA-DR dot-plot to exclude B lymphocytes^21^.

### RNA extraction and microarray assay

Peripheral venous blood samples were collected prior to the caesarian section in EDTA-treated tubes. CD14++ and CD16++ monocytes were isolated using magnetic sorting as described above and immediately lysed in 1 mL QIAZOL reagent for the following RNA extraction according to the manufacturer's instructions (Qiagen). Biotinylated cRNA was prepared according to the standard Affymetrix protocol. Following fragmentation, 10 μg of cRNA were hybridized for 16 h at 45 °C on GeneChip™ Human Gene 2.0 ST Array. GeneChips were washed and stained in the Affymetrix Fluidics Station 450. GeneChips were scanned using the Affymetrix GeneChip Scanner 3000 7g. The data were analyzed with Transcriptome Analysis Console (TAC) Software version 4.0.2 using Affymetrix default analysis settings and RMA algorithm.

### Enrichment analysis

Panther (Protein ANalysis THrough Evolutionary Relationships) gene classification analysis was used for relating gene sequence to specific molecular functions, biological processes, and pathways^39^. Fisher's exact test with FDR correction was performed for estimation of statistical over-representation.

### Real-time polymerase chain reaction (RT-PCR) and gene expression assay

Total RNA was isolated from CD14++ cells using QIAZOL (Qiagen); the corresponding cDNA was synthesized by using the MMLV-RT kit (Evrogen, Russia). The polymerase chain reactions were set in triplicates with qPCRmix-HS SYBR master mixes containing Sybr Green I dye (Evrogen, Russia). The target-specific primers (Table S4 in Supplementary Materials) were designed by using the Primer-BLAST tool (NCBI, USA) in accordance with the general rules and custom ordered from Evrogen. Expression was quantified by the threshold cycle (Ct) approach; the relative expression levels were evaluated via an algorithm proposed by M. Pfaffl with *B2M* as a housekeeping reference target^54^.

### Statistical analysis

The data is presented as mean ± standard error mean (SEM) and mean ± standard deviation (SD). The Shapiro–Wilk test was used to estimate the normality of distributions. One-way analysis of variance (ANOVA) followed by Tukey’s post-hoc test was used to identify differences among multiple groups with normal distribution, while the Kruskal–Wallis non-parametric ANOVA followed by the post-hoc Dunn test was used to calculate statistical differences for non-normal distributions. Data from two groups were compared by Mann–Whitney test or *t* test depending on the distribution. All calculations were performed by Prism 7.0 software (GraphPad, USA). p-value < 0.05 was considered significant i.e. indicative for differences in comparison to the control.

## Supplementary Information

Supplementary Information.

## Data Availability

This study did not generate new unique reagents. The original contributions presented in the study are included in the article/supplementary files; further inquiries can be directed to the corresponding author. The data reported in this paper have been deposited into the Gene Expression Omnibus (GEO) database, https://www.ncbi.nlm.nih.gov/geo (GEO accession: GSE186819).
