# Author Correction: Distinct gene expression patterns for CD14++ and CD16++ monocytes in preeclampsia

**DOI:** 10.1038/s41598-022-23677-w

**Published:** 2022-11-07

**Authors:** Polina Vishnyakova, Maria Kuznetsova, Anastasiya Poltavets, Mariia Fomina, Viktoriia Kiseleva, Kamilla Muminova, Alena Potapova, Zulfiya Khodzhaeva, Alexey Pyregov, Dmitry Trofimov, Andrey Elchaninov, Gennady Sukhikh, Timur Fatkhudinov

**Affiliations:** 1 National Medical Research Center for Obstetrics, Gynecology and Perinatology Named After Academician V.I. Kulakov of Ministry of Healthcare of Russian Federation, Moscow, Russia; 2 Peoples’ Friendship University of Russia, Moscow, Russia; 3 A.P. Avtsyn Research Institute of Human Morphology, Moscow, Russia

Correction to: *Scientific Reports* 10.1038/s41598-022-19847-5, published online 14 September 2022

The original version of this Article contained errors. The names of the authors Polina Vishnyakova, Maria Kuznetsova, Anastasiya Poltavets, Mariia Fomina, Viktoriia Kiseleva, Kamilla Muminova, Alena Potapova, Zulfiya Khodzhaeva, Alexey Pyregov, Dmitry Trofimov, Andrey Elchaninov, Gennady Sukhikh and Timur Fatkhudinov were incorrectly given as Vishnyakova Polina, Kuznetsova Maria, Poltavets Anastasiya, Fomina Mariia, Kiseleva Viktoriia, Muminova Kamilla, Potapova Alena, Khodzhaeva Zulfiya, Pyregov Alexey, Trofimov Dmitry, Elchaninov Andrey, Sukhikh Gennady and Fatkhudinov Timur. Consequently, the initials of the author names were also reversed in the Author Contributions section.

The original Article has been corrected.

