# Supplementary material for: Distinct gene expression patterns for CD14++ and CD16++ monocytes in preeclampsia
Source: Sci Rep. 2022 Sep 14;12:15469. doi: 10.1038/s41598-022-19847-5 (PMC9474473; doi:10.1038/s41598-022-19847-5)
Supplement: Supplementary file 1 — Supplementary Information. [file 41598_2022_19847_MOESM1_ESM.docx]

**Supplementary Material**

**Table S1. Top 10 up-regulated and down-regulated transcripts in loPE CD14++ monocytes vs CD14++ monocytes of the control patients.**

| Up-regulated | Fold Change | P-val | Down-regulated | Fold Change | P-val |
| --- | --- | --- | --- | --- | --- |
| linc-FCGR1B-7 | 34.4 | 3.3E-06 | ADGRE4P | -20.32 | 0.0003 |
| linc-RAB23-2 | 23.99 | 3.3E-05 | TRAJ35 | -5.33 | 0.0315 |
| linc-PARD6G-2 | 9.37 | 2.7E-06 | TRAV20 | -4.59 | 0.0008 |
| linc-POTED-9 | 7.6 | 7.5E-06 | IL7R | -3.14 | 0.0118 |
| CXCL8 | 5.71 | 6.8E-03 | TRBV3-1 | -3.12 | 0.0035 |
| JUN | 4.47 | 1.7E-03 | ARL17A | -3.07 | 0.0314 |
| CXCR1 | 4.15 | 8.1E-03 | TRAJ39 | -3.02 | 0.0012 |
| RPS2P46 | 4.08 | 1.2E-03 | ITK | -2.6 | 0.0045 |
| TNFAIP6 | 3.61 | 1.2E-03 | TC2N | -2.53 | 0.0076 |
| U1 spliceosomal RNA | 3.56 | 1.7E-03 | SLAMF6 | -2.51 | 0.006 |

**Table S2. Enrichment analysis (results of overrepresentation) of genes significantly down-regulated in CD14++Mo of loPE patients vs control group performed in PANTHER.**

| Biological Process | Signaling pathway | fold enrichment | P-value | FDR |
| --- | --- | --- | --- | --- |
|  | Negative T cell selection (GO:0043383) | 62.79 | 2.64E-05 | 1.97E-02 |
|  | T cell selection (GO:0045058) | 23.36 | 3.60E-05 | 2.17E-02 |
|  | Regulation of natural killer cell mediated immunity (GO:0002715) | 20.5 | 5.80E-05 | 3.03E-02 |
|  | Positive regulation of interferon-gamma production (GO:0032729) | 16.1 | 1.98E-05 | 1.55E-02 |
|  | T cell differentiation (GO:0030217) | 14.77 | 1.60E-08 | 6.27E-05 |
|  | T cell receptor signaling pathway (GO:0050852) | 13.45 | 7.47E-06 | 8.37E-03 |
|  | Regulation of interferon-gamma production (GO:0032649) | 12.88 | 9.49E-06 | 9.91E-03 |
|  | T cell activation (GO:0042110) | 10.67 | 9.47E-09 | 4.95E-05 |
|  | Positive regulation of innate immune response (GO:0045089) | 9.79 | 4.21E-05 | 2.45E-02 |
|  | Lymphocyte differentiation (GO:0030098) | 8.76 | 1.12E-06 | 1.76E-03 |
|  | Regulation of lymphocyte mediated immunity (GO:0002706) | 8.66 | 8.11E-05 | 3.85E-02 |
|  | Regulation of lymphocyte differentiation (GO:0045619) | 8.33 | 1.00E-04 | 4.61E-02 |
|  | Mononuclear cell differentiation (GO:1903131) | 7.61 | 3.44E-06 | 4.50E-03 |
|  | Regulation of leukocyte mediated immunity (GO:0002703) | 7.45 | 5.10E-05 | 2.86E-02 |
|  | Lymphocyte activation (GO:0046649) | 7.33 | 1.05E-07 | 2.73E-04 |
|  | Regulation of innate immune response (GO:0045088) | 7.15 | 6.58E-05 | 3.22E-02 |
|  | Leukocyte differentiation (GO:0002521) | 6.26 | 1.58E-05 | 1.38E-02 |
|  | Adaptive immune response (GO:0002250) | 6.24 | 5.71E-09 | 4.48E-05 |
|  | Leukocyte activation (GO:0045321) | 5.78 | 1.22E-06 | 1.74E-03 |
|  | Activation of immune response (GO:0002253) | 5.58 | 1.06E-04 | 4.60E-02 |
|  | Positive regulation of immune response (GO:0050778) | 5.55 | 6.59E-07 | 1.15E-03 |
|  | Cell activation (GO:0001775) | 4.69 | 1.01E-05 | 9.87E-03 |
|  | Positive regulation of immune system process (GO:0002684) | 4.57 | 1.54E-07 | 3.45E-04 |
|  | Hemopoiesis (GO:0030097) | 4.56 | 3.13E-05 | 2.14E-02 |
|  | Hematopoietic or lymphoid organ development (GO:0048534) | 4.22 | 6.25E-05 | 3.16E-02 |
|  | Regulation of cytokine production (GO:0001817) | 4.13 | 3.41E-05 | 2.14E-02 |
|  | Regulation of immune response (GO:0050776) | 4.09 | 3.52E-06 | 4.25E-03 |
|  | Immune system development (GO:0002520) | 3.98 | 1.05E-04 | 4.70E-02 |
|  | Immune response (GO:0006955) | 3.68 | 3.28E-08 | 1.03E-04 |
|  | Regulation of immune system process (GO:0002682) | 3.64 | 1.86E-07 | 3.64E-04 |
|  | Immune system process (GO:0002376) | 3.33 | 5.75E-10 | 9.01E-06 |

**Table S3. Top 10 up-regulated and down-regulated transcripts in loPE CD16++ monocytes vs CD16++ monocytes of the patients from the control group.**

| Up-regulated | Fold Change | P-val | Down-regulated | Fold Change | P-val |
| --- | --- | --- | --- | --- | --- |
| linc-RAB23-2 | 20.73 | 0.0007 | OLR1 | -4.08 | 0.0297 |
| linc-PARD6G-2 | 19.15 | 0.005 | linc-BOLA2 | -3.74 | 1.75E-06 |
| linc-FCGR1B-7 | 12.49 | 0.0036 | SNORD56B | -3.86 | 0.0012 |
| linc-POTED-9 | 9.58 | 0.0006 | lnc-TMOD3-1 | -3.52 | 0.0024 |
| ALAS2 | 6.28 | 0.0001 | U6 small nuclear RNA 522 | -3.42 | 5.66E-05 |
| HLA-B | 4 | 0.0055 | U7 small nuclear 195 pseudogene | -3.35 | 0.0049 |
| HBA1 | 3.11 | 0.0028 | U6 spliceosomal RNA | -3.33 | 0.0109 |
| TRAJ9 | 2.97 | 0.0125 | lnc-RBM17-3 | -3.06 | 0.0002 |
| linc-ZNF25-4 | 2.95 | 0.006 | U6 small nuclear RNA 942 | -3.04 | 0.0008 |
| GTF2H2 | 2.89 | 0.0378 | U6 small nuclear RNA 60 | -2.98 | 0.0097 |
| linc-NBPF6-2 | 2.76 | 0.0471 | ADGRE4P | -2.88 | 0.0168 |
| MT-TL2 | 2.75 | 0.0189 | linc-COBL-1 | -2.85 | 0.0037 |

**Table S4. Primer sequences.**

| Gene | 5`-3` | Product size |
| --- | --- | --- |
| CXCR1_F | ATC TTC CTG CTT TGC TGG CT | 264 |
| CXCR1_R | GGT AAC ACG ATG ACG TGC CA |  |
| JUN_F | CAG CCA GGT CGG CAG TAT AG | 281 |
| JUN_R | GGA CTC TGC CAC TTG TCT CC |  |
| CXCL8_F | AGT CCT TGT TCC ACT GTG CC | 490 |
| CXCL8_R | CAC AGC ACT ACC AAC ACA GC |  |
| ADGRE4P_F | GCT CTC ATG TGC ACA GCA AC | 204 |
| ADGRE4P_R | AGG TGC TGG TGT TCT GGA TG |  |
| IL7R_F | ACG ATG TAG CTT ACC GCC AG | 228 |
| IL7R_R | TAG GAT CCA TCT CCC CTG AGC |  |
| ARL17A_F | GTC GGC GGT GTT GTA GGT AG | 223 |
| ARL17A_R | TGT CTC CAC ACA GAA ACC TAC TG |  |

**
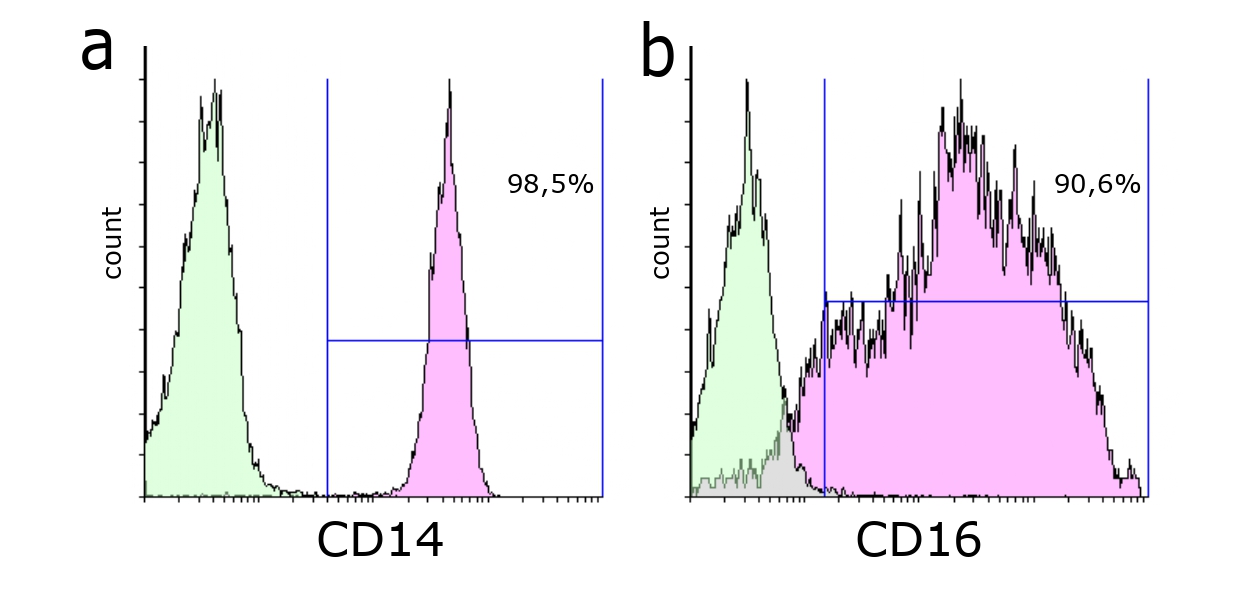
**

**Fig S1. Representative histograms of two cells` fractions obtained after sorting with CD14 (a) and CD16 (b) beads stained with antibodies to CD14 and CD16 (pink), respectively, and the staining control (green).**

**
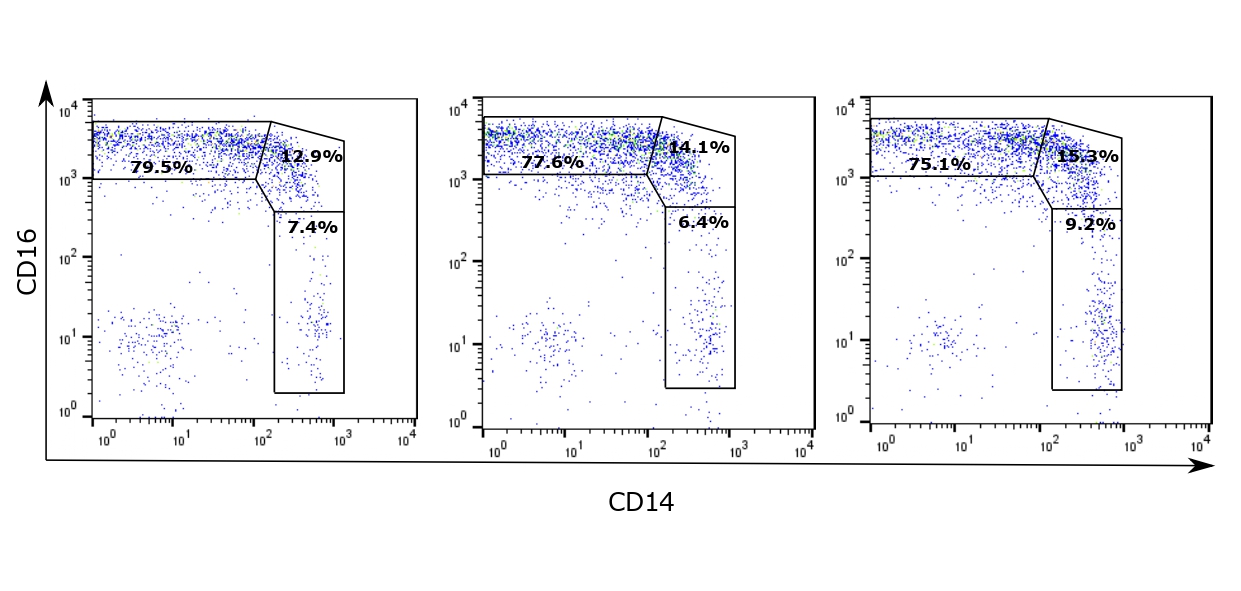
**

**Fig S2. Representative CD14-CD16 dot-plots showed anti-CD14 and anti-CD16 staining of monocyte fractions after CD16 sorting.**
